# Large-scale epidemiological modelling: scanning for mosquito-borne diseases spatio-temporal patterns in Brazil

**DOI:** 10.1098/rsos.241261

**Published:** 2025-05-28

**Authors:** Eduardo C. Araujo, Cláudia T. Codeço, Sandro Loch, Luã B. Vacaro, Laís Picinini Freitas, Raquel M. Lana, Leonardo S. Bastos, Iasmim F. de Almeida, Fernanda Valente, Luiz Max Carvalho, Flávio C. Coelho

**Affiliations:** ^1^ School of Applied Mathematics, Getulio Vargas Foundation, Rio de Janeiro, Brazil; ^2^ Programa de Computação Científica, FIOCRUZ, Rio de Janeiro, Brazil; ^3^ École de Santé Publique, Université de Montréal, Montreal, Quebec, Canada; ^4^ Centre de Recherche en Santé Publique, Montreal, Quebec, Canada; ^5^ Barcelona Supercomputing Center, Barcelona, Spain; ^6^ Department of Epidemiology, Escola Nacional de Saúde Pública Sergio Arouca, Rio de Janeiro, Rio de Janeiro, Brazil; ^7^ Observatório de Bioeconomia, FGV são paulo, São Paulo, São Paulo, Brazil

**Keywords:** Episcanner, mosquito-borne diseases, climate-epidemic nexus, geographical and temporal patterns

## Abstract

The influence of climate on mosquito-borne diseases like dengue and chikungunya is well established, but comprehensively tracking long-term spatial and temporal trends across large areas has been hindered by fragmented data and limited analysis tools. This study presents an unprecedented analysis, in terms of breadth, estimating the susceptible-infectious-recovered transmission parameters from incidence data in all 5570 municipalities in Brazil over 14 years (2010–2023) for both dengue and chikungunya. We describe the Episcanner computational pipeline, developed to estimate these parameters, producing a reusable dataset characterizing all dengue and chikungunya epidemics that have taken place in this period in Brazil. The analysis reveals new insights into the climate-epidemic nexus: we identify distinct geographical and temporal patterns of arbovirus disease incidence across Brazil, highlighting how climatic factors like temperature and precipitation influence the timing and intensity of dengue and chikungunya epidemics. The innovative Episcanner tool empowers researchers and public health officials to explore these patterns in detail, facilitating targeted interventions and risk assessments. This research offers the possibility of exploring the main characteristics of dengue and chikungunya epidemics and their geographical specificities linked to the effects of global temperature fluctuations such as those captured by the El Niño-Southern Oscillation index.

## 1. Introduction

Dengue and chikungunya are among the most significant mosquito-borne diseases in terms of disease burden and potential for global expansion. Both are arboviruses; the first one contemplates four serotypes (DENV-1−4) and the second, the predominant lineage, is the East/Central/South African genotype (ECSA) [1,2]. These diseases are transmitted by *Aedes aegypti*, the primary vector, and *Aedes albopictus* [2,3]. Worldwide, the estimated number of dengue cases has increased from 30 million, in 1990, to 56 million in 2019 [1], reaching more than 100 countries. Furthermore, chikungunya has been spreading worldwide since the early 2000s and is now present in all continents with tropical zones [4].

The temporal dynamics of these vector-borne diseases are characterized by seasonal and multi-year cycles that can vary from place to place. Within seasons, they can differ further, as evidenced by key parameters such as the basic reproductive number ($R_{0}$), which represents the average number of new cases generated by a single infected individual in a susceptible population; the peak size, which indicates the maximum magnitude of cases during an outbreak; the timing, which refers to when the peak occurs over a season or cycle; and the total attack rate, which refers to the cumulative proportion of the population that has been infected during the epidemic period [5–7]. This spatial-temporal heterogeneity is strongly associated with the climate and meteorological conditions that affect the mosquito vectorial capacity and viral transmission, in combination with the history of previous population exposure that affects the collective immunity level. The urban environment, characterized by crowding and inadequate waste and water services, can further increase the population vulnerability and exposure, while effective vector control measures can potentially flatten the curve. Consequently, these local factors collectively contribute to the emergence of highly diverse spatio-temporal epidemic dynamics.

Dengue has maintained a continuous presence in the tropical regions of Brazil since 1986 [8], with patterns varying from transient transmission to epidemic and endemic persistence [5]. Chikungunya fever was introduced in Brazil in 2014 and persisted, with outbreaks increasing in frequency [9]. In the last years, probably owing to the gradual warming of the southern states, outbreaks of both diseases have begun to occur in areas with subtropical and temperate climates [2,3]. Hence, understanding how climate can affect the reproductive number and timing of dengue and chikungunya epidemics locally is useful for guiding decisions at the national level.

This study is centred on estimating and examining the characteristics of dengue and chikungunya epidemics that occurred in Brazilian municipalities from 2010 to 2023. The key descriptors are the reproductive number, epidemic size and peak week, estimated by the Episcanner pipeline, which is an optimized workflow that structures a sequence of steps for data processing, model training and prediction generation developed for this end.

## 2. Material and methods

### 2.1. Data sources

Brazil comprises 5570 municipalities, divided into 26 states, plus the Federal District, the country’s capital. The disease data for all the municipalities were obtained from the Infodengue project [10], available at info.dengue.mat.br, for the period between 2010 and 2023. The Infodengue project gathers notification data for dengue, chikungunya and zika from the Brazilian Ministry of Health, sanitizes and re-publishes it, along with epidemiological analyses, such as estimates for the effective reproduction number, $R_{t}$, by week, for all municipalities. The $R_{t}$ is defined as the mean number of secondary cases generated by a single index case throughout its infectious period following [11]. This metric quantifies disease transmissibility and was calculated according to the methodology outlined in [12]. The code for obtaining the Infodengue data is available on the GitHub repository associated with this paper. The climate data were obtained from the Copernicus ERA5 re-analysis dataset [13] and daily averaged by the municipality. The climate variables included land-surface temperature, relative humidity, precipitation and atmospheric pressure.

### 2.2. The Episcanner pipeline

If all municipalities experienced a dengue epidemic per year, in 10 years, there would be 55 700 epidemics, varying in velocity, timing and magnitude. This is a large number of models to be fitted individually. Thus, Episcanner is a computational pipeline that efficiently scans all these potential epidemics and estimates the epidemiological descriptors whenever an epidemic is detected. The epidemic descriptors are the basic reproduction number ($R_{0}$), peak week, the total outbreak size, as well as timing and duration of the epidemic.

The core of the Episcanner pipeline is the Richards logistic growth function, a sigmoid curve that has a one-to-one relationship with the susceptible-infectious-recovered (SIR) epidemiological model [14]. Based on this relationship, the epidemiologically meaningful parameters of the SIR transmission model can be efficiently derived from the fitted Richard model, which serves as a curve template for identifying an epidemic. This simplification is pivotal for curtailing the computational cost generated by estimating the epidemic descriptors for 5570 municipalities during 14 years. The Richards model has been used in the analysis of dengue epidemics, as shown in [15]. Additionally, the methodology for estimating SIR parameters through the Richards model has been applied to dengue data, as presented in [14].

**Figure 1.**
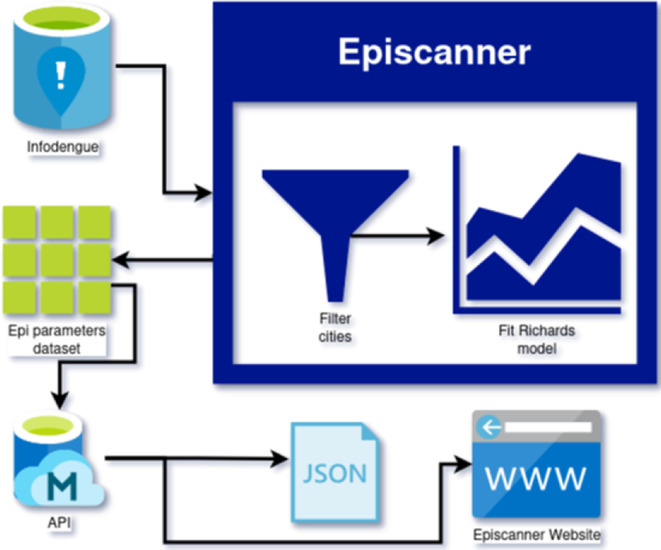
The computational pipeline of the Episcanner inferential engine is the dark blue block. Data from Infodengue, after being filtered to identify candidates for epidemic years, is then fitted to the Richards model. The resulting estimated parameter dataset is made available through an API as a JavaScript object notation (JSON) object (https://api.mosqlimate.org/datastore/). These data can be visualized in the Episcanner dashboard, which is part of the Infodengue website (https://info.dengue.mat.br/epi-scanner).

The Episcanner pipeline is depicted in figure 1 and can be summarized in the following steps:

(i) Infodengue’s incidence time series are obtained from their application programming interface (API);
(ii) filter years with a potential *epidemic*, defined as an annual time series having a minimum of three weeks with at least 0.9 probability of $R_{t}>1$ and more than 50 cumulative cases. Any year with less than that is discarded as a short outbreak. This is done for all municipalities;
(iii) split all-time series at week 45 to obtain a collection of 52 week long time series, one for each year and each municipality. For each year y in which an epidemic was detected, the Richards model was applied to the time window between week 45 of y−1 and week 45 of y, a period of 52 weeks. This cut point was chosen as it is the typical beginning of the dengue season in Brazil;
(iv) the Richards model is fitted to the observed curves using an optimization algorithm, generating the SIR parameter estimates (see below for details); and
(v) epidemiological parameters dataset is created and made available through the Mosqlimate API.

The complete set of parameters produced from all Brazilian municipalities can be browsed through the online dashboard https://info.dengue.mat.br/epi-scanner/ called Episcanner, within the Infodengue website. This dashboard is updated weekly and allows anyone to browse the estimated epidemic characteristics of dengue and chikungunya, as described in this paper.

### 2.3. Estimation of the epidemiological parameters

The Richards model is defined by the following equation:


(2.1) $$\begin{array}{lrr} & \begin{array}{r} J(t)=L-L[1+\alpha e^{b(t-t_{j})}{]}^{-1/\alpha }, \end{array} & \end{array}$$

where J(t) is interpreted as the accumulated number of cases at week t, L>0 is the estimated total number of cases at the end of the epidemic and $t_{j}\in \left[1,52\right]$ is the week of the inflection point of the sigmoid curve, which corresponds to the peak of the epidemic. The remaining parameters α and b are additional parameters to be estimated.

When mapping to the SIR model (details available in the electronic supplementary material), the function J(t) corresponds to the sum of the number of recovered and infected individuals at time t, which in the SIR model is denoted as R(t)+I(t). The equivalent SIR model’s parameters can be derived from the Richards equation’s parameters (equation (2.1)) through the equations given below (refer to [14] for derivation):


(2.2)β=bα,(2.3)γ=b(1α−1),(2.4)R0=βγ=11−α.

**Figure 2.**
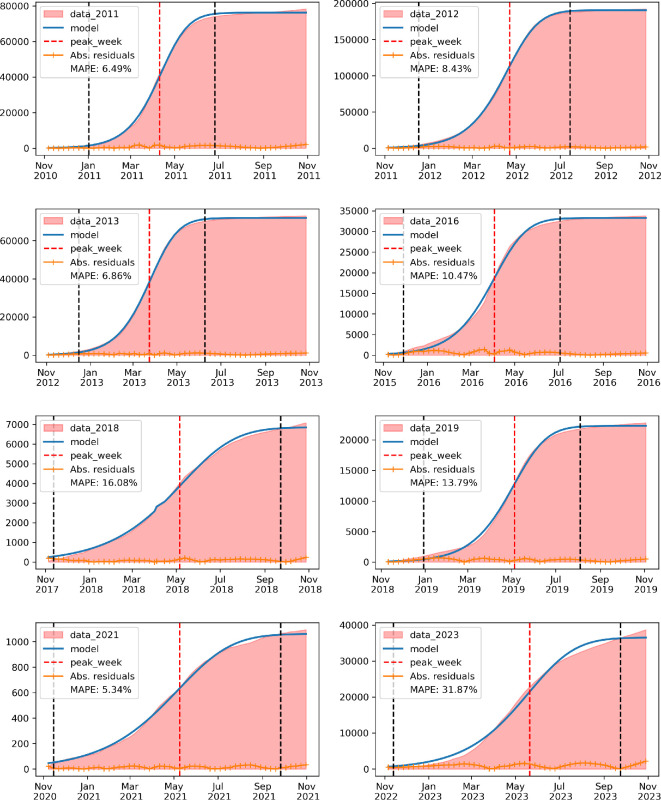
Example of the outcome of estimating the start and end weeks of an epidemic for Rio de Janeiro municipality in all epidemic years between 2010 and 2023. The red area represents the cumulative number of cases. Vertical black dashed lines indicate the start and end of the epidemic, and the vertical red dashed line represents the estimated peak week. The blue curve is the fitted Richards curve, and the orange one at the bottom is the absolute error of the fit every week. The final text in the legend denotes the mean absolute percentage error (MAPE) of the fitted curve.

Besides the reproductive number ($R_{0}$), epidemic size (L) and peak week ($t_{j}$), the Episcanner dataset also includes the onset of the epidemic ($w_{s}$) that is defined as the week in which the new cases crossed the 5th percentile, and the final week of the epidemic ($w_{e}$) defined as the week when the cases dropped below this threshold (see figure 2). From these points, the epidemic duration ($w_{e}-w_{s}$) in weeks is computed and also added to the dataset.

From now on, we will refer to these derived parameters, collectively, as *epidemic parameters* or *epidemic descriptors*.

### 2.4. Fitting the model to data

For every selected city and epidemic year, as defined in §2.2, we fitted the Richards model equation (2.1) to the data by solving the following optimization problem: determining the values of the parameters set $\xi :=\left\{L,\alpha ,b,t_{j}|L,\alpha ,b,t_{j}\right\}$*,* all of which are real and positive, that minimizes the error function below:


(2.5) $$\begin{array}{lrr} & \begin{array}{r} {\mathrm{argmin}}_{\xi }\;\sum_{t=1}^{\tau }\frac{(C(t)-J(t,\xi ){)}^{2}}{\tau }, \end{array} & \end{array}$$

where C(t) is the observed cumulative number of cases at week t and τ=52. The following ranges restricted the possible values of the parameters of ξ: γ∈[0.95,1.05] (one week infectious period), α∈[0.001,1]*,* $b\in \left[10^{-6},1\right]$, $t_{j}\in \left[5,35\right]$ (based on the overall distribution of peak weeks). Additionally, we restricted the possible values of α to always satisfy $\alpha =\frac{b}{\gamma +b}$ in equation (2.3) [14]. The parameters were restricted to avoid the overfitting problem described in [14] and get biologically reasonable values for the parameters estimated.

This fitting process is computationally more efficient than fitting the SIR ordinary differential equations, since it avoids numerical integration steps. The optimization was implemented with the *lmfit* Python package,^1^ using a global optimization approach based on the differential evolution algorithm [16]. The algorithm was chosen for offering the optimal balance between computational efficiency and fitting quality among the algorithms available within the package, with the default configurations being used; more details can be found in [17]. The Richards model does not include spatial dependence between the cities because of greater computational demand from including a spatial component. However, a regression model is later proposed to explore the association between the estimated epidemiological parameters and climate variables across time and space, in which spatial dependence is incorporated.

### 2.5. Predicting the week of epidemic peak

One question of interest is how early the epidemic peak will occur in a given year and place. To test if the dengue epidemic descriptors, estimated by Episcanner, show a correlation to epidemiological, demographic and climate variables, we built a histogram gradient boosting regression (HGBR) model using the implementation provided by the *Scikit-learn* Python package.^2^ This model was selected for its computational efficiency, particularly in terms of runtime performance on large datasets. Given our feature matrix consisting of 31 variables, it exhibited improved efficiency compared to conventional gradient boosting regressors. Additionally, it outperforms alternative models, such as random forests, in terms of $R^{2}$ score [18]. The model outcome is the peak week of epidemics, $W_{i,y}^{peak}$for every epidemic year in every location. Predictors included lagged climate variables, case counts and population sizes, as well as lagged epidemic descriptors. The regression model proposed can be written as below:


(2.6)Wi,ypeak∼Ci,y−1+Di,y−1+Ei,y−1+Si+ε,

where $W_{i,y}^{peak}$ is the peak week of the epidemic at municipality i on year y. $C_{i,y-1}$*,* $D_{i,y-1}$ and $E_{i,y-1}$ are respective sets of climate, demographic and epidemiological predictors. The climate predictors include the aggregation of temperature, humidity, precipitation and El Niño-Southern Oscillation (ENSO) index in different time windows. The only demographic predictor used was the population size of the last year. Epidemiological parameters include the number of cases, peak week and reproduction number from the previous year. The latitude and longitude values of each municipality were used as spatial predictors, represented by $S_{i}$ in the model. The term ε denotes the random error component of the model having a Gaussian distribution with mean zero. A comprehensive description of all model features is provided in the electronic supplementary material, table S1. The incorporation of epidemiological and climatic predictors into the model is warranted by the observed reduction in prediction error, as demonstrated in the electronic supplementary material, table S2. Moreover, as indicated by equation (2.6), only a 1 year lag was used. Longer lags were not considered, as they would render the models unfeasible for municipalities with shorter data series.

The model was fitted using data encompassing all Brazilian cities for the entire period, filtered as described above for epidemic parameter estimation. A single model was fitted to each geographical region of Brazil. Brazil has five geographical regions, namely, north (N), northeast (NE), midwest (MW), southeast (SE) and south (S). This model was fitted only for dengue because of its broader geographical coverage. We used the shapley additive explanations (SHAP values) framework [19] to analyse the importance of each feature (predictor) of the model. The SHAP scale quantifies the contribution of each feature to the model’s output. Positive SHAP values indicate an increase in the predicted outcome, whereas negative values signify a decrease. This metric provides a comprehensive understanding of the influence of individual predictors on model predictions, enhancing interpretability and facilitating more informed decision-making.

## 3. Results

### 3.1. Spatio-temporal patterns of dengue and chikungunya epidemics

Of the 5570 Brazilian cities, 4096 (73%) had at least one epidemic year for dengue and 1263 cities (22%) for chikungunya between 2010 and 2023. There were 18 567 dengue epidemic events and 2199 chikungunya epidemic events. Table 1 contains summary statistics for some of the parameters estimated. The full set of parameters also includes the SIR model’s β and γ parameters, the α parameter from the Richards equation, and the starting and ending weeks of the epidemic.

**Table 1.** Summary statistics for selected parameters estimated for dengue and chikungunya across all epidemics.

	$R_{0}$	duration	peak week	total cases	incidence (per 100k)
**dengue**					
mean	1.97	26.62	22.04	992.09	2146.22
s.d.	0.52	10.38	5.80	4262.09	2841.39
25%	1.60	19.00	18.40	113.75	523.45
50%	1.86	25.00	22.11	240.40	1203.41
75%	2.20	33.00	25.24	624.70	2626.13
**chikungunya**					
mean	2.12	25.47	24.69	624.43	1156.76
s.d.	0.70	12.17	7.29	2341.62	1620.65
25%	1.60	16.0	19.78	98.65	213.80
50%	1.94	24.0	24.68	195.33	594.58
75%	2.45	34.0	29.51	461.99	1436.29

**Figure 3.**
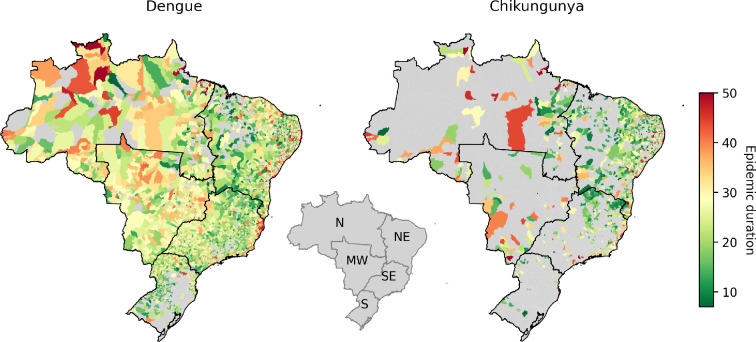
Median epidemic duration (in weeks) estimated for dengue and chikungunya cases. Note the increase in the duration of dengue epidemics as one moves from the southern to the northern states. This trend is less marked but also visible for chikungunya epidemics. In the smaller map in the middle, we have the geographical regions of Brazil: north (N), northeast (NE), midwest (MW), southeast (SE) and south (S).

The spatial distribution of the estimated epidemic parameters/quantities ($R_{0}$, peak week and epidemic duration) exhibits discernible patterns across different regions of Brazil. Notably, the average duration of dengue epidemics exhibits an ascending gradient from the SE to the northwest (NW regions; see figure 3. The reproductive numbers of both dengue and chikungunya exhibited spatial and temporal variability across the country (see electronic supplementary material, figure S1). This variability is evident between different geographical regions and over time.

**Figure 4.**
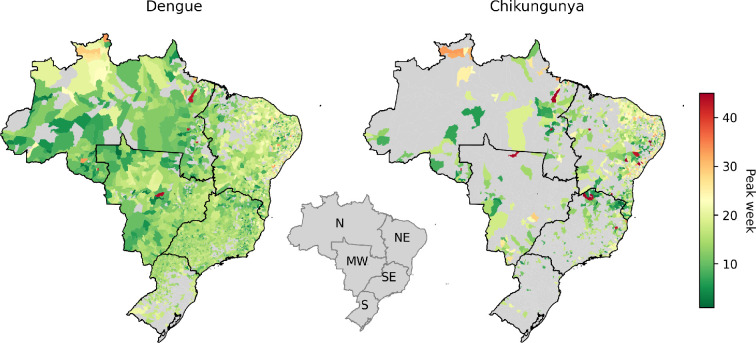
Median peak weeks estimated for dengue and chikungunya epidemics between 2010 and 2023 by municipality. The inset map shows Brazil's geographic regions: north (N), northeast (NE), midwest (MW), southeast (SE) and south (S). The peak values were rescaled, as they were estimated using a window starting in week 45, to ensure that these values represent the peak week within the full annual range of weeks 1−52.

**Figure 5.**
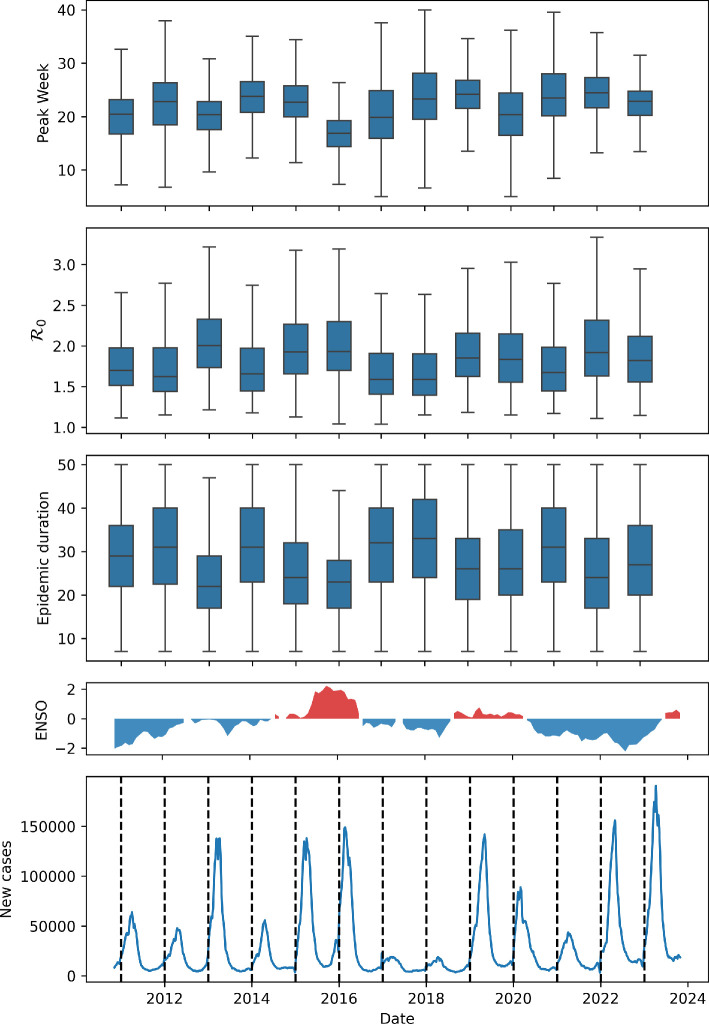
The boxplots, from top to bottom, show the estimated peak week, the estimated basic reproduction number and the epidemic duration in weeks, for dengue epidemics by year. The last panel is the time series of dengue cases in Brazil between November 2010 and November 2023. Above it, is the ENSO index time series, coloured red for El Niño, and blue for La Niña periods.

The estimated duration of dengue epidemics varied from short (<10 weeks) to year-long. Longer epidemics were seen mostly in the north region (figure 3). The peak week tends to occur earlier for dengue than chikungunya and in the west earlier than the east side of the country (see figure 4). Looking at the country-wide time series for dengue, we can see that higher incidence peaks are associated with shorter epidemics with higher $R_{0}$ (figure 5). The decrease in dengue cases in 2017 and 2018 may be linked to the significant increase in chikungunya cases. As a disease recently introduced into Brazil, chikungunya had one of the highest incidence rates until 2017, the second year with the highest number of cases recorded between 2014 and 2022. During this period, there may have been under-reporting of dengue cases as chikungunya, owing to similar symptoms and the scarcity of specific diagnostic tests. In addition, the simultaneous presence of chikungunya, dengue and zika may have generated epidemiological competition in which infection by one arbovirus provides temporary protection against others [20,21].

**Figure 6.**
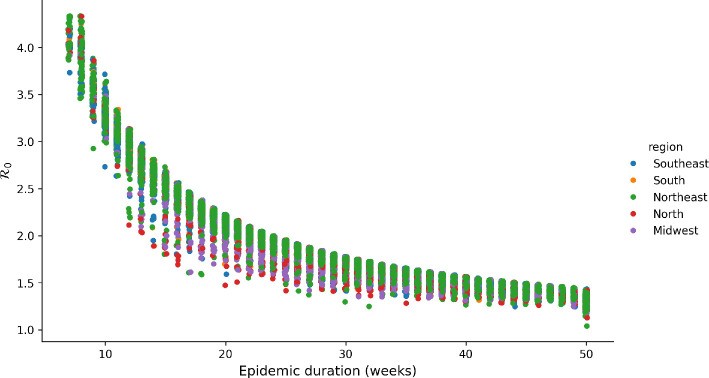
Estimated duration of the dengue epidemic as a function of estimated $R_{0}$. Longer epidemics are associated with lower estimates for $R_{0}$. Dots are individual cities.

A nonlinear relationship was seen between epidemic duration and $R_{0}$ (see figure 6). Epidemics with high $R_{0}$ tend to be shorter, while outbreaks with low $R_{0}$ tend to last longer. This trend was also discernible, albeit less prominently, in the context of chikungunya epidemics, see the electronic supplementary material, figure S2.

### 3.2. Earlier epidemics and climate

**Figure 7.**
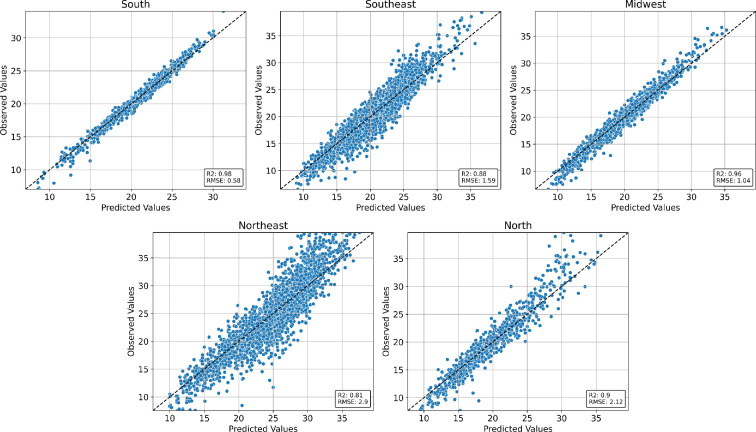
Performance in-sample of the histogram gradient boosting regression model trained with the entire dataset. Each point represents the predicted versus observed weeks for a city. The dashed black line represents the identity.

Figure 7 depicts the HGBR model’s performance in predicting the dengue epidemic peak week as a function of climate and epidemiological covariates measured 1 year before. The model provided more accurate predictions in the south, southeast and midwest, where seasonality is stronger. The root mean squared error (RMSE) in the north region (RMSE = 2.12) is higher compared to the south (RMSE = 0.58), southeast (RMSE = 1.59) and midwest (RMSE = 1.04). Nevertheless, this predictive error is still smaller than the historical variation in the peak week for the region, of ±4–7 weeks.

**Figure 8.**
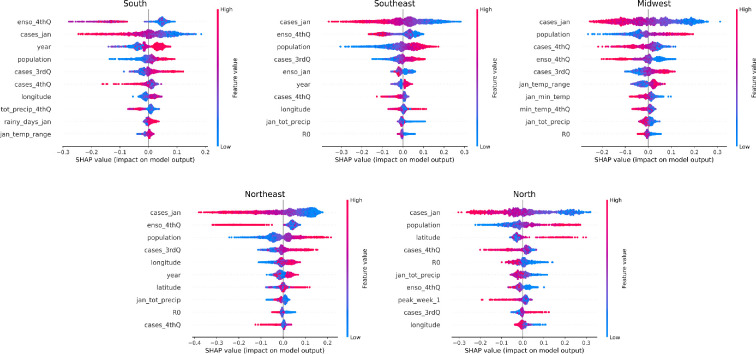
The top 10 most important predictors in each region, selected from all predictors listed in the electronic supplementary material, table S1, encoded as SHAP values. The SHAP scale represents how much each feature affects the model's output (peak week), with positive values associated with an increase in peak week and negative with a decrease. Each dot represents one epidemic. Their colour is mapped to the value of each feature, with blue standing for lower values and pink for higher. The 0 in the SHAP scale is aligned with the expected value of each feature above. The suffix ‘4thQ’ indicates the features computed in the fourth quarter of the previous year, the suffix ‘3rdQ’ indicates the features computed in the fourth quarter of the previous year, the label ‘jan’ indicates the features computed during January of the current year. If there is no label, with the exception of ‘year’, the feature was computed during the previous year.

When we look at the importance of the model’s features (figure 8), in all regions, the features related to the *population* and *cases* in the previous year were always among the top 10 most important features. Also, all regions had at least two climate predictors in the top 10, with the average ENSO value during the fourth quarter of the previous year being the most common among them.

**Figure 9.**
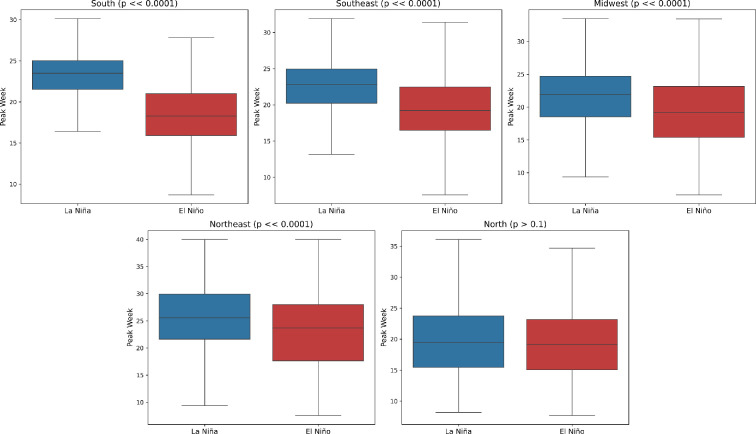
Estimated median peak week of dengue epidemics by region for El Niño and La Niña years. La Niña years, in this analysis, are years when the mean ENSO index in the last quarter of the previous year was below 0, and El Niño years are the ones where the mean ENSO index in the last quarter of the year was above 0. The *p*-values for each region are provided in the subplot title.

The ENSO index stood out as an important feature to explain variations in the timing of epidemics. ENSO is a climate phenomenon measuring the sea surface temperature (SST) of the Pacific Ocean over time. It has three phases or states: periods with above average SST are called El Niño, periods with below average temperatures are called La Niña. The third state is neutral, when SST are close to average. We looked specifically at the value of the ENSO index in the last quarter of the previous year. After performing a *t*‐test on the peak samples under different ENSO conditions, we found that positive ENSO values (El Niño) in the fourth quarter were associated with earlier epidemics in the following year in all regions except the north (figure 9). By contrast, negative ENSO values (La Niña) during the fourth quarter were linked to later epidemics in the south, northeast, midwest and southeast regions (*p* **<<** 0.0001). No statistically significant difference was found in the north region for the peak values under these conditions (*p* = 0.16).

## 4. Discussion

This study introduces a computational pipeline (figure 1) capable of processing incidence data from all municipalities and estimating key epidemic statistics in a standardized format. To achieve the required computational scalability, we employed optimization techniques to fit the Richards model, which has a one-to-one relationship with the archetypical SIR mathematical model. Even though the SIR model is an approximation to the transmission mechanisms taking place in the field, we believe that this simplified description of the epidemics is a small price to pay in exchange for developing a consistent set of epidemic parameters over a large spatio-temporal range. Using more detailed transmission models would be undesirable, because of the added computational cost to fit and the added uncertainty associated with the estimation of a larger number of parameters subject to identifiability issues [22,23].

Following parameter estimation, a regression analysis was conducted to elucidate the influence of local and global climate patterns on epidemic parameters. Although not exhaustive, these exploratory analyses revealed a discernible association between the ENSO and epidemic timing (figure 9), represented by the peak week of the epidemic in four of the five regions. The regression analysis results show that various epidemiological, demographic and climatic factors determine the shape of future epidemics. This association has been reported before for Singapore [24], Thailand [25] and other regions of the world [26–29]. However, our study differentiates itself by the larger spatio-temporal scale of the analysis and for generating an open dataset that enables further explorations and will be continuously updated.

The observed association between $R_{0}$ and epidemic duration, shown in figure 6, exhibits remarkable consistency, partly reflecting the interdependence between these parameters within the transmission model. Given the environmental influences driving epidemics, for example, the well-described correlation between transmission and temperature, it is noteworthy that these two parameters remain highly correlated, as per theoretical expectations, that assume constant transmission rates and endogenous determination of duration (by depletion of susceptibles). The small size of the epidemics (relative to population size) suggests that such depletion is not to be expected, except in the hypothesis of a massive under-reporting of cases in most cities, which could mask larger attack ratios.

The extensive dataset of epidemiological parameters generated by Episcanner has demonstrated utility in developing predictive models, particularly when integrated with complementary datasets such as climate and demographic series. Although predictive modelling was only superficially explored in this study, its potential for future research is considerable. To our knowledge, this dataset represents a unique large-scale compilation of epidemic parameters estimated using a consistent methodology, enabling robust comparisons. We anticipate that statistical modellers will use this open dataset for future investigations.

## 5. Conclusion

The methodological framework introduced in this paper facilitates the comprehensive characterization of epidemic dynamics across both temporal and spatial dimensions. This approach offers invaluable insights into the transmission patterns of arbovirus diseases, enabling public health authorities to devise timely interventions to mitigate disease spread effectively. Furthermore, the identification of spatial variations in epidemic parameters underscores the imperative for region-specific strategies in disease surveillance and control initiatives.

The predictive potential for this parameter set has been demonstrated and can be further elaborated to reveal more specific associations at smaller spatial scales. One limitation of such models is the lack of data on the immunological status of the population, i.e. the proportion of the population previously exposed to each dengue serotype. Data about the proportion of cases infected with each DENV type per week or at least per year would contribute significantly to understanding these epidemics long-term dynamics. We believe that making such data available would be the most important governmental investment in controlling arbovirus diseases.

The epidemiological parameters used in this paper are updated weekly for the current year and are made available through Episcanner, a publicly available web dashboard https://info.dengue.mat.br/epi-scanner/.

Continuous monitoring of the epidemiological characteristics (described in §2.3) of dengue and chikungunya can be a key tool for efficient control of their incidence and morbidity. When it comes to accurate epidemiological assessments, methodological consistency over time and geographical space is indispensable. In particular, for robust comparisons across time and space, employing the same analytical methods is crucial to ensure that methodological differences are not confounded with data variations.

This study elucidates longer-term association patterns between dengue epidemic intensity and global climate variations, as exemplified by the multivariate ENSO index. These findings reinforce the longstanding hypothesis [30] that the ongoing escalation in global average temperatures will inevitably exacerbate the burden of mosquito-borne diseases.

## Data Availability

Data and relevant code for this research work are stored in GitHub [31] and have been archived within the Zenodo repository [32]. Supplementary material is available online [33].

## Footnotes

^1^

https://lmfit.github.io/lmfit-py/intro.html

^2^

https://scikit-learn.org

## References

[1] Yang X, Quam MBM, Zhang T, Sang S. 2021 Global burden for dengue and the evolving pattern in the past 30 years. J. Travel Med. **28**, taab146. (10.1093/jtm/taab146) 34510205

[2] de Almeida IF *et al*. 2023 The expansion of chikungunya in Brazil. Lancet Reg. Health Am. **25**, 100571. (10.1016/j.lana.2023.100571) 37638140 PMC10448323

[3] Codeco CT *et al*. 2022 Fast expansion of dengue in Brazil. Lancet Reg. Health Am. **12**, 100274. (10.1016/j.lana.2022.100274) 36776428 PMC9904033

[4] Manzoor KN *et al*. 2022 The global emergence of Chikungunya infection: an integrated view. Rev. Med. Virol. **32**, e2287. (10.1002/rmv.2287) 34428335

[5] de Almeida IF, Lana RM, Codeço CT. 2022 How heterogeneous is the dengue transmission profile in Brazil? A study in six Brazilian states. PLoS Neglected Trop. Dis. **16**, 1–20. (10.1371/journal.pntd.0010746) PMC9499305 36095004

[6] Tabataba FS, Chakraborty P, Ramakrishnan N, Venkatramanan S, Chen J, Lewis B, Marathe M. 2017 A framework for evaluating epidemic forecasts. BMC Infect. Dis. **17**, 1–27. (10.1186/s12879-017-2365-1) 28506278 PMC5433189

[7] Valencia VAN, Díaz Y, Pascale JM, Boni MF, Sanchez-Galan JE. 2023 Using compartmental models and particle swarm optimization to assess dengue basic reproduction number R0 for the Republic of Panama in the 1999-2022 period. Heliyon **9**, e15424. (10.1016/j.heliyon.2023.e15424) 37128312 PMC10147988

[8] Rodriguez-Barraquer I, Cordeiro MT, Braga C, de Souza WV, Marques ET, Cummings DAT. 2011 From re-emergence to hyperendemicity: the natural history of the dengue epidemic in Brazil. PLoS Neglected Trop. Dis. **5**, e935. (10.1371/journal.pntd.0000935) PMC3014978 21245922

[9] de Souza WM *et al*. 2023 Spatiotemporal dynamics and recurrence of chikungunya virus in Brazil: an epidemiological study. Lancet Microbe **4**, e319–e329. (10.1016/s2666-5247(23)00033-2) 37031687 PMC10281060

[10] Codeco C, Coelho F, Cruz O, Oliveira S, Castro T, Bastos L. 2018 Infodengue: a nowcasting system for the surveillance of arboviruses in Brazil. Revue d’Épidémiologie et de Santé Publique **66**, S386. (10.1016/j.respe.2018.05.408)

[11] Cowling BJ, Lau MSY, Ho LM, Chuang SK, Tsang T, Liu SH, Leung PY, Lo SV, Lau EHY. 2010 The effective reproduction number of pandemic influenza. Epidemiology **21**, 842–846. (10.1097/ede.0b013e3181f20977) 20805752 PMC3084966

[12] Codeço CT, Villela DAM, Coelho FC. 2018 Estimating the effective reproduction number of dengue considering temperature-dependent generation intervals. Epidemics **25**, 101–111. (10.1016/j.epidem.2018.05.011) 29945778

[13] Muñoz Sabater J, Service CCC. 2019 ERA5-land hourly data from 1950 to present. See https://cds.climate.copernicus.eu/.

[14] Wang XS, Wu J, Yang Y. 2012 Richards model revisited: validation by and application to infection dynamics. J. Theor. Biol. **313**, 12–19. (10.1016/j.jtbi.2012.07.024) 22889641

[15] Sanna M, Wu J, Zhu Y, Yang Z, Lu J, Hsieh YH. 2018 Spatial and temporal characteristics of 2014 dengue outbreak in Guangdong, China. Sci. Rep. **8**, 2344. (10.1038/s41598-018-19168-6) 29402909 PMC5799376

[16] Storn R, Price K. 1997 Differential evolution–a simple and efficient heuristic for global optimization over continuous spaces. J. Glob. Optim. **11**, 341–359. (10.1023/A:1008202821328)

[17] Project AD. 2024 Episcanner-downloader: downloader for epidemiological scanners. See https://github.com/AlertaDengue/episcanner-downloader (accessed 11 November 2024).

[18] Scikit-learn developers. 2024 HistGradientBoostingRegressor — scikit-learn documentation. See https://scikit-learn.org/stable/modules/generated/sklearn.ensemble.HistGradientBoostingRegressor.html (accessed 10 February 2025).

[19] Lundberg SM, Lee SI. 2017 A unified approach to interpreting model predictions. Adv. Neural Inf. Process. Syst. **30**.https://proceedings.neurips.cc/paper_files/paper/2017/file/8a20a8621978632d76c43dfd28b67767-Paper.pdf

[20] da Saúde M. 2023 Boletins Epidemiológicos - Arboviroses no Brasil. See https://www.gov.br/saude/pt-br/assuntos/boletins-epidemiologicos (accessed 12 November 2024).

[21] Mota ML *et al*. 2021 Serological and molecular epidemiology of the Dengue, Zika and Chikungunya viruses in a risk area in Brazil. BMC Infect. Dis. **21**, 1–7. (10.1186/s12879-021-06401-3) 34303348 PMC8310596

[22] Gallo L, Frasca M, Latora V, Russo G. 2022 Lack of practical identifiability may hamper reliable predictions in COVID-19 epidemic models. Sci. Adv. **8**, eabg5234. (10.1126/sciadv.abg5234) 35044820 PMC8769547

[23] Kao YH, Eisenberg MC. 2018 Practical unidentifiability of a simple vector-borne disease model: implications for parameter estimation and intervention assessment. Epidemics **25**, 89–100. (10.1016/j.epidem.2018.05.010) 29903539 PMC6264791

[24] Earnest A, Tan SB, Wilder-Smith A. 2012 Meteorological factors and El Niño Southern Oscillation are independently associated with dengue infections. Epidemiol. Infect. **140**, 1244–1251. (10.1017/s095026881100183x) 21906411

[25] Tipayamongkholgul M, Fang CT, Klinchan S, Liu CM, King CC. 2009 Effects of the El Niño-Southern Oscillation on dengue epidemics in Thailand, 1996-2005. BMC Public Health **9**, 1–15. (10.1186/1471-2458-9-422) 19930557 PMC2785791

[26] Johansson MA, Cummings DAT, Glass GE. 2009 Multiyear climate variability and dengue—El Niño Southern Oscillation, weather, and dengue incidence in Puerto Rico, Mexico, and Thailand: a longitudinal data analysis. PLoS Med. **6**, e1000168. (10.1371/journal.pmed.1000168) 19918363 PMC2771282

[27] Moraes BCd, Souza EBd, Sodré GRC, Ferreira DBdS, Ribeiro JBM. 2019 Sazonalidade nas notificações de dengue das capitais da Amazônia e os impactos do El Niño/La Niña. Cad. Saúde Pública **35**, e00123417. (10.1590/0102-311x00123417) 31531519

[28] Banu S, Guo Y, Hu W, Dale P, Mackenzie JS, Mengersen K, Tong S. 2015 Impacts of El Niño Southern Oscillation and Indian Ocean Dipole on dengue incidence in Bangladesh. Sci. Rep. **5**, 16105. (10.1038/srep16105) 26537857 PMC4633589

[29] Do TTT, Martens P, Luu NH, Wright P, Choisy M. 2014 Climatic-driven seasonality of emerging dengue fever in Hanoi, Vietnam. BMC Public Health **14**, 1–10. (10.1186/1471-2458-14-1078) 25323458 PMC4287517

[30] Reiter P. 2001 Climate change and mosquito-borne disease. Environ. Health Perspect. **109**, 141–161. (10.1289/ehp.01109s1141) 11250812 PMC1240549

[31] eduardocorrearaujo. 2025 repo_episcanner_paper. GitHub. See https://github.com/eduardocorrearaujo/repo_episcanner_paper.

[32] Araujo EC, Coelho FC. 2024 eduardocorrearaujo/repo_episcanner_paper: large-scale epidemiological modeling: scanning for mosquito-borne diseases spatio-temporal patterns in Brazil (v1.0.0). Zenodo. (10.5281/zenodo.13236547) PMC12115816 40438543

[33] Araujo EC. 2025 Supplementary material from: Large-scale epidemiological modeling: scanning for mosquito-borne diseases. Figshare. (10.6084/m9.figshare.c.7826788) PMC12115816 40438543

